# Supplementary material for: The Influence of BMP6 on Serotonin and Glucose Metabolism
Source: Int J Mol Sci. 2024 Jul 18;25(14):7842. doi: 10.3390/ijms25147842 (PMC11276723; doi:10.3390/ijms25147842)
Supplement: Supplementary file 1 [file ijms-25-07842-s001.zip › ijms-3089725-supplementary.pdf]

Rat primers used for gene expression analysis of INS-1 832/13 cell supernatant samples		
**Gene**	**Forward primer (5'-3')**	**Reverse primer (5'-3')**
Phosphoenolpyruvate carboxykinase *(Pepck)*	GCGCAAGTACTCTGTGTGGA	TGAAAGGCCGCACCATGTAT
Glucose transporter 2 *(Glut2)*	CAGCTGTCTCTGTGCTGCTTGT	GCCGTGATGCTCACATAACTGA
Glucokinase *(Gck)*	CACCCCAGAAGGCTCAGAAG	AGCATCACTCTGAAGTTGGTACC
Glucose 6-phosphatase *(G6Pase)*	TTGTGGTTGGGATACTGGGC	ATGCCTGACAAGACTCCAGC
Insulin *(Ins)*	CTACAGTCGGAAACCATCAGCA	CCACCAAGTGACAACCACAAAG
Serotonin transporter *(5htt)*	TCTGAAAAGCCCCACTGGACT	TAGGACCGTGTCTTCATCAGGC
Tryptophan hydroxylase-1 *(Tph1)*	AGCATAACCAGCGCCATGAA	GGCATCATTGACGACATCGAG
5-Hydroxytryptamine receptor 2B *(5ht2b)*	GGCTGATTTGCTGGTTGGATTG	GGGCCATGTAGCCTCAAACATG
β-actin	GCGCAAGTACTCCTGTGTGGA	ACATCRGCTGGAAGGTGGAC
**Mouse primers used for gene expression analysis of homogenized tissue samples**		
**Gene**	**Forward primer (5'-3')**	**Reverse primer (5'-3')**
Phosphoenolpyruvate carboxykinase *(Pepck)*	AAATGCTTTGCGTTGCGGAT	CGGGGTTAGTTATGCCCAGG
Glucose transporter 2 *(Glut2)*	TGTTGGGGCCATCAACATGA	ACATGCCAATCATCCCGGTT
Glucokinase *(Gck)*	CACCCCAGAAGGCTCAGAAG	TCTAGACGGACTCAGCACAAA
Glucose 6-phosphatase *(G6Pase)*	GAGCGCAACAGTTCCCTTTG	ACCAAAGACTCTGCATGCCT
Serotonin transporter (*5htt*)	CTCATCTTCACCATTATCTACTTCAG	CTCACCAGCAGGACAGAAAG
Tryptophan hydroxylase-1 *(Tph1)*	CACGAGTGCAAGCCAAGGTTT	AGTTTCCAGCCCCGACATCAG
5-Hydroxytryptamine receptor 2B *(5ht2b)*	AACGCCTAACACGGTGGACTG	CCAACTGAGGACATTCTTCGCATAAG
5-Hydroxytryptamine receptor 2C *(5ht2c)*	GCCCCGTCTGGATTTCACTA	TTGCTACATACCGGTCCAGC
Forkhead box protein O1 *(Foxo1)*	GCTCTGTGCGCCTAAGTACA	AGCTGGCTGGTTTCCAAGTT
Glyceraldehyde 3-phosphate dehydrogenase *(Gapdh)*	AGGTCGGTGTGAACGGATTTG	TGTAGACCATGTAGTTGAGGTCA

Table S1. Primers used for gene expression analysis via RT-qPCR

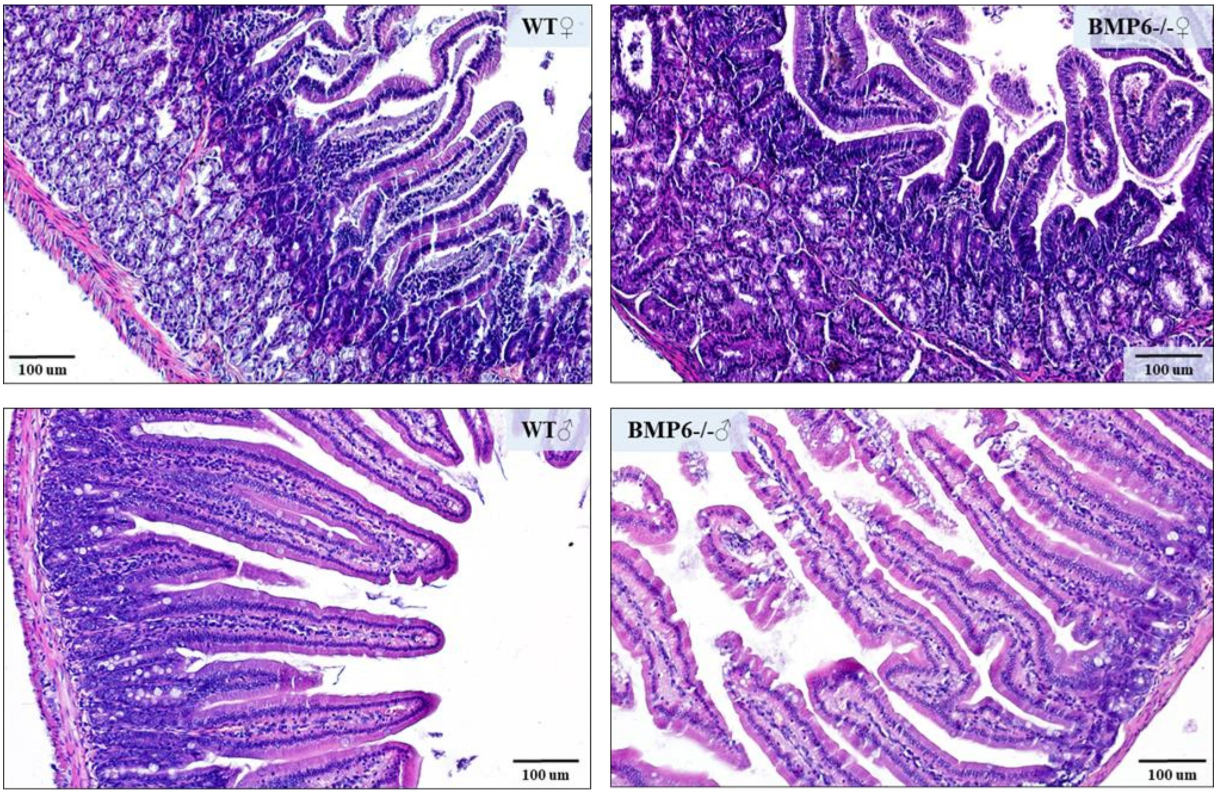

Figure S1. H&E staining of the duodenum of female and male WT and BMP6-/- mice, magnicifation 20×, scale bar = 100 μm (n=3).

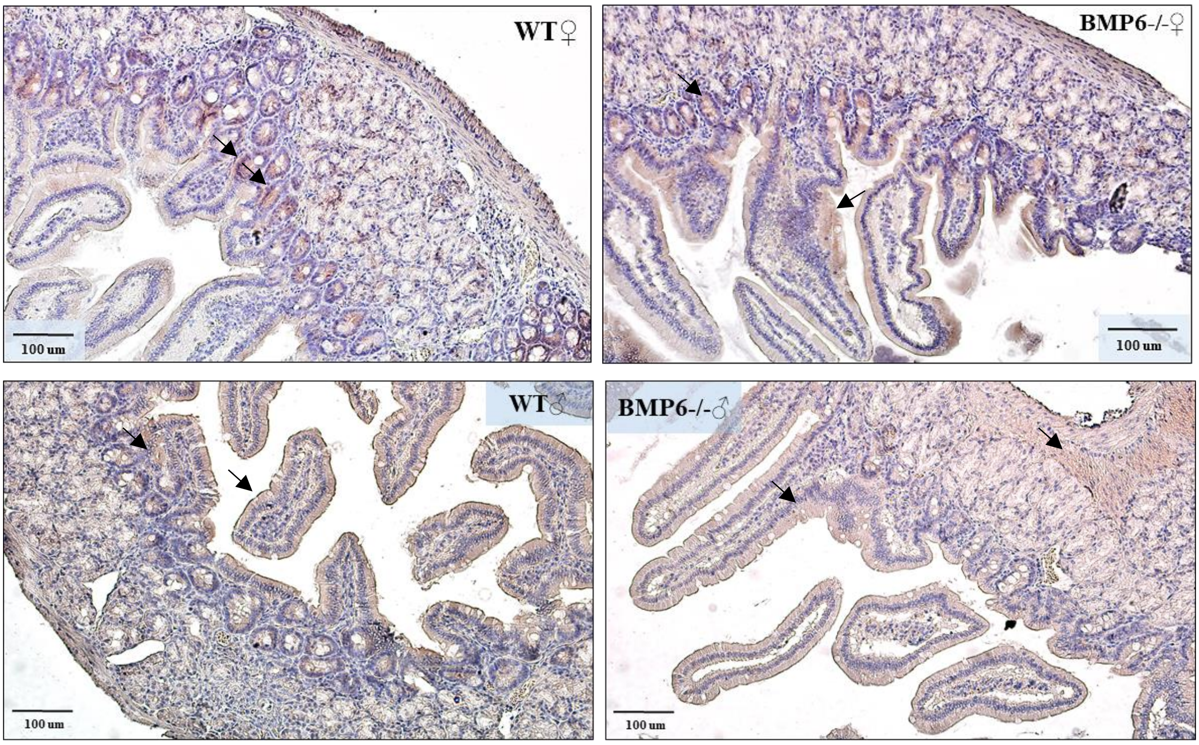

Figure S2. Immunohistochemical analysis of 5HTT (red color) in the duodenum from female and male WT and BMP6-/- mice, magnification 20×, scale bar = 100 μm (n=3). 5HTT is primarily localized in the epithelial cells of the mucosal layer of the duodenum (black arrows).

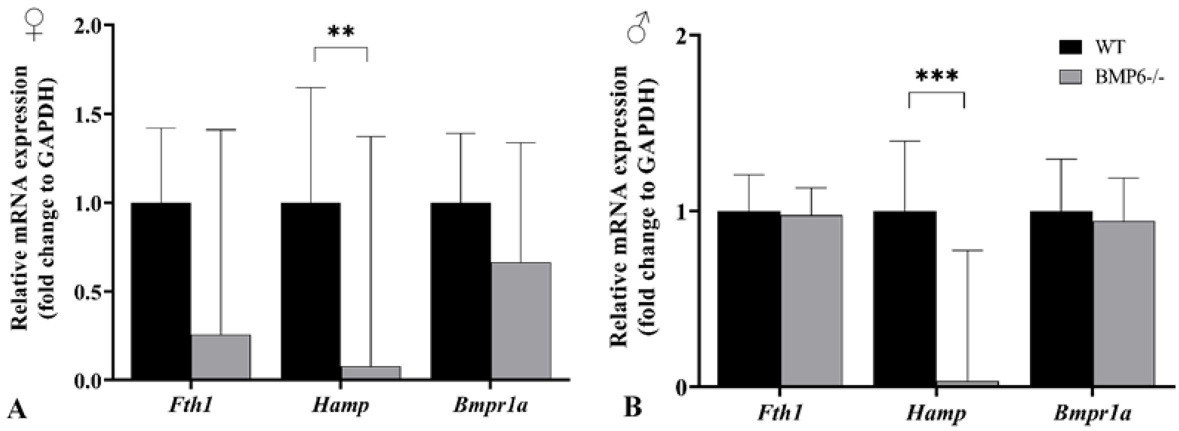

Figure S3. Relative mRNA expression of iron metabolism-related genes (*Fth1* and *Hamp*) and a gene for bone morphogenetic protein receptor type 1A (*Bmpr1a*) was measured with RT-qPCR in the liver of (A) female, and (B) male BMP6-/- and WT mice. Results are reported as fold change + SEM, $^{**}p<0.01$ $^{***}p<0.001$.

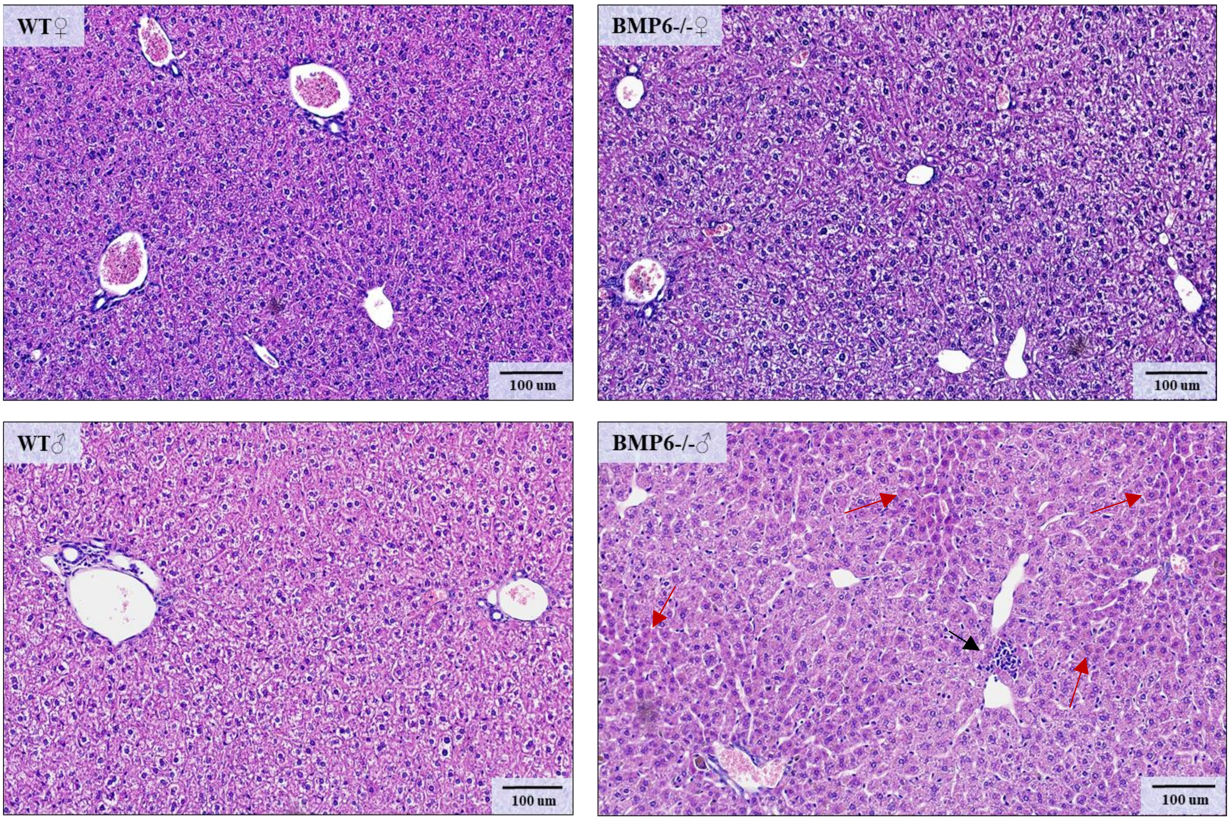

Figure S4. H&E staining of the liver of female and male WT and BMP6-/- mice, magnification 20×, scale bar = 100 μm (n=3). No histological differences were observed in the liver between female WT and BMP6-/- mice, while in the male mice BMP6-/- were observed signs of leukocyte infiltration in the sinusoids (black arrow), and iron accumulation within hepatocytes (brown pigment, red arrows).
